# Brain activity changes after high/low frequency stimulation in a nonhuman primate model of central post-stroke pain

**DOI:** 10.1038/s41598-024-67440-9

**Published:** 2024-07-17

**Authors:** Kazuaki Nagasaka, Noriyuki Higo

**Affiliations:** 1 Human Informatics and Interaction Research Institute, National Institute of Advanced Industrial Science and Technology, 1-1-1 Umezono, Tsukuba, Ibaraki 305-8568 Japan; 2 Institute for Human Movement and Medical Sciences, Niigata University of Health and Welfare, 1398 Shimami-Cho, Kita-Ku, Niigata, 950-3198 Japan; 3 Department of Physical Therapy, Niigata University of Health and Welfare, 1398 Shimami, Kita-Ku, Niigata, 950-3198 Japan

**Keywords:** Allodynia, Hyperalgesia, Neuromodulation, Thalamic pain, Stroke, Chronic pain, Neuroscience, Neurology

## Abstract

Central post-stroke pain (CPSP) is a chronic pain resulting from a lesion in somatosensory pathways. Neuromodulation techniques, such as repetitive transcranial magnetic stimulation (rTMS) that target the primary motor cortex (M1), have shown promise for the treatment of CPSP. High-frequency (Hf) rTMS exhibits analgesic effects compared to low-frequency (Lf) rTMS; however, its analgesic mechanism is unknown. We aimed to elucidate the mechanism of rTMS-induced analgesia by evaluating alterations of tactile functional magnetic resonance imaging (fMRI) due to Hf- and Lf-rTMS in a CPSP monkey model. Consistent with the patient findings, the monkeys showed an increase in pain threshold after Hf-rTMS, which indicated an analgesic effect. However, no change after Lf-rTMS was observed. Compared to Lf-rTMS, Hf-rTMS produced enhanced tactile-evoked fMRI signals not only in M1 but also in somatosensory processing regions, such as the primary somatosensory and midcingulate cortices. However, the secondary somatosensory cortex (S2) was less active after Hf-rTMS than after Lf-rTMS, suggesting that activation of this region was involved in CPSP. Previous studies showed pharmacological inhibition of S2 reduces CPSP-related behaviors, and the present results emphasize the involvement of an S2 inhibitory system in rTMS-induced analgesia. Verification using the monkey model is important to elucidate the inhibition system.

## Introduction

Central post-stroke pain (CPSP) is a chronic pain that occurs several weeks after a cerebrovascular accident in the somatosensory pathway, including the posterolateral thalamus and lateral medulla^1–6^. Patients with CPSP experience spontaneous and induced pain, such as allodynia and hyperalgesia. Pharmacological treatment provides limited benefit for these symptoms^7^.

Multiple neuroimaging findings in patients with CPSP suggest that the pathophysiological mechanism underlying CPSP may be a reorganization of pain-related networks and increased brain activity in component regions resulting from late-onset maladaptive plasticity after stroke^3,8–11^. In this context, non-pharmacological approaches such as neurostimulation and neuromodulation have been attempted in patients with CPSP. Repetitive transcranial magnetic stimulation (rTMS) is a non-invasive brain stimulation technique that stimulates the cortex using electromagnetic induction^12^. High-frequency rTMS (Hf-rTMS; pulse stimulation at frequencies ≥ 5 Hz) targeting the primary motor cortex (M1) has produced promising outcomes for various pain symptoms, including CPSP^10,13–15^. In contrast, low-frequency rTMS (Lf-rTMS; pulse stimulation at frequencies ≤ 1 Hz) targeting the M1 produces an insufficient analgesic effect in healthy individuals and patients with pain^15–17^. Traditionally, Hf-rTMS increases cortical excitability, whereas Lf-rTMS has the opposite effect^18,19^. These facts suggest that stimulus frequency affects CPSP-related brain activity differentially. However, the mechanism linking activity changes to pain relief is yet to be established. Currently, the M1 rTMS efficacy is approximately 40%, and its effects last for hours to days^15,20^. No clear cumulative effect was identified in a study with a 10-day intervention period^13^. To maximize the efficacy and duration of rTMS, understanding the activity changes after Hf-rTMS is required.

To address these issues, we used our established CPSP macaque model^21^, which exhibits increased brain activity in pain-related brain regions, such as the primary somatosensory cortex (S1), secondary somatosensory cortex (S2), posterior insular cortex (PIC), and anterior cingulate cortex (ACC), in response to non-invasive tactile stimuli^22^, consistent with the findings in patients with CPSP^3,10^. Using the CPSP macaque model, Kadono et al. reported that M1 targeting by Hf-rTMS (5 Hz) showed analgesic effects and functional connectivity between the pain-related regions was restored to the level observed before the stroke^23^. However, this study evaluated brain activity in the resting state, as measured by functional magnetic resonance imaging (fMRI); thus, the effect of high and low rTMS on sensory-evoked brain responses is yet to be examined. Given that the impact of brain stimulation differs significantly with neural state^24,25^, identifying the sensory-induced fMRI signal changes, which may vary with different analgesic effects of Hf-rTMS and Lf-rTMS, is a pivotal step toward elucidating the analgesic mechanisms.

## Results

### Lesion area and behavioral change after rTMS

**Figure 1:**
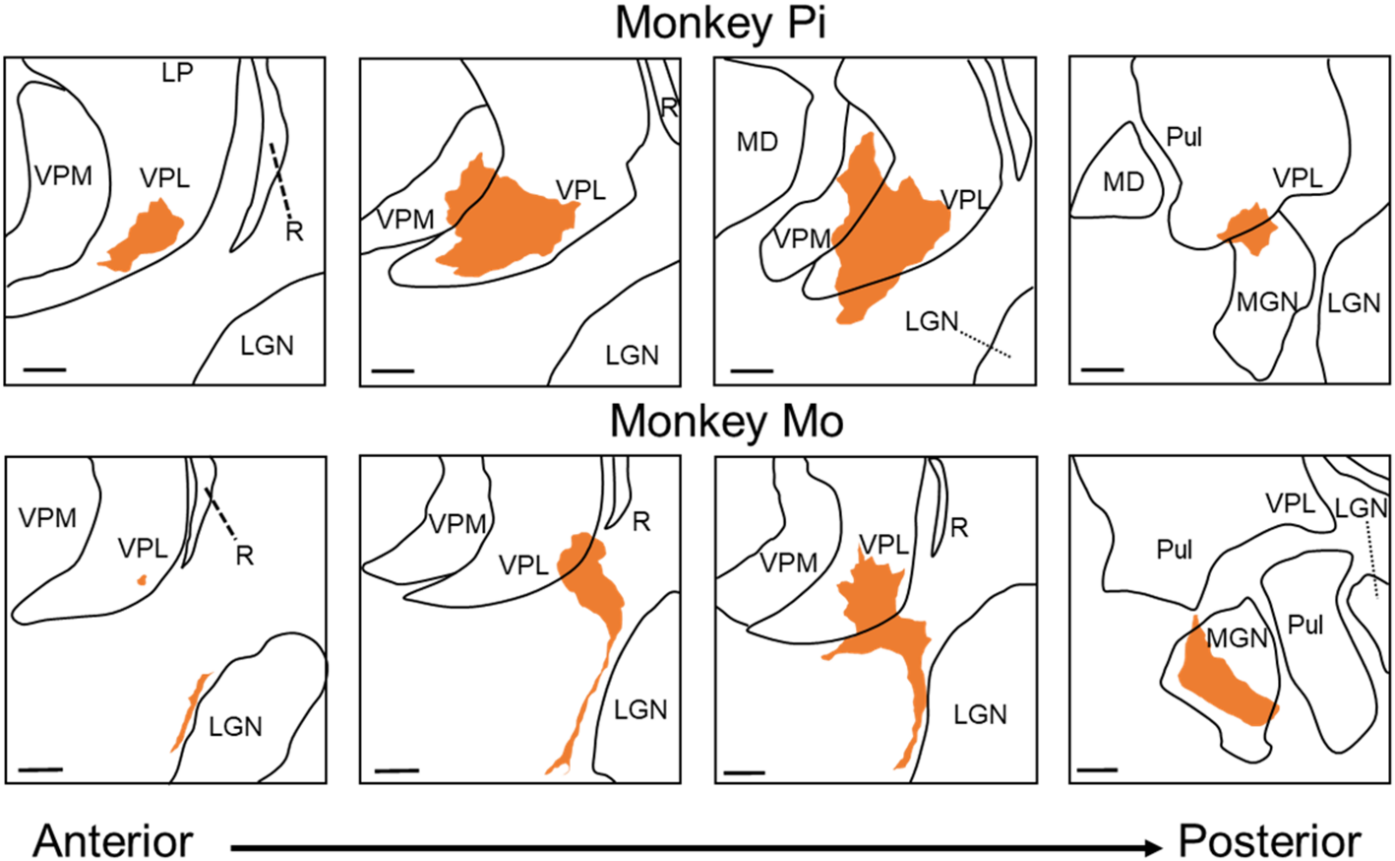
Confirmation of lesional area. Line drawing of the left thalamus in each lesioned monkey. Serial coronal images, spaced by approximately 500–600 μm, are arranged from anterior (left) to posterior (right). Orange color shading indicates the lesion areas. LP, lateral posterior nucleus; MD, mediodorsal nucleus; Pul, pulvinar nucleus; VPM, ventral posteromedial nucleus; VPL, ventral posterolateral nucleus; LGN, lateral geniculate nucleus; MGN, medial geniculate nucleus; R, reticular thalamus. Scale bar = 1 mm.

Histological verification confirmed that the lesion areas included the thalamic ventral posterolateral nucleus (VPL) in both monkeys; in Monkey Pi, the lesions extended to the ventral posteromedial nucleus (VPM) and medial geniculate nucleus (MGN). In contrast, the lesions extended to the MGN, lateral geniculate nucleus, and adjacent white matter regions in Monkey Mo^26^ (Fig. 1).

**Figure 2:**
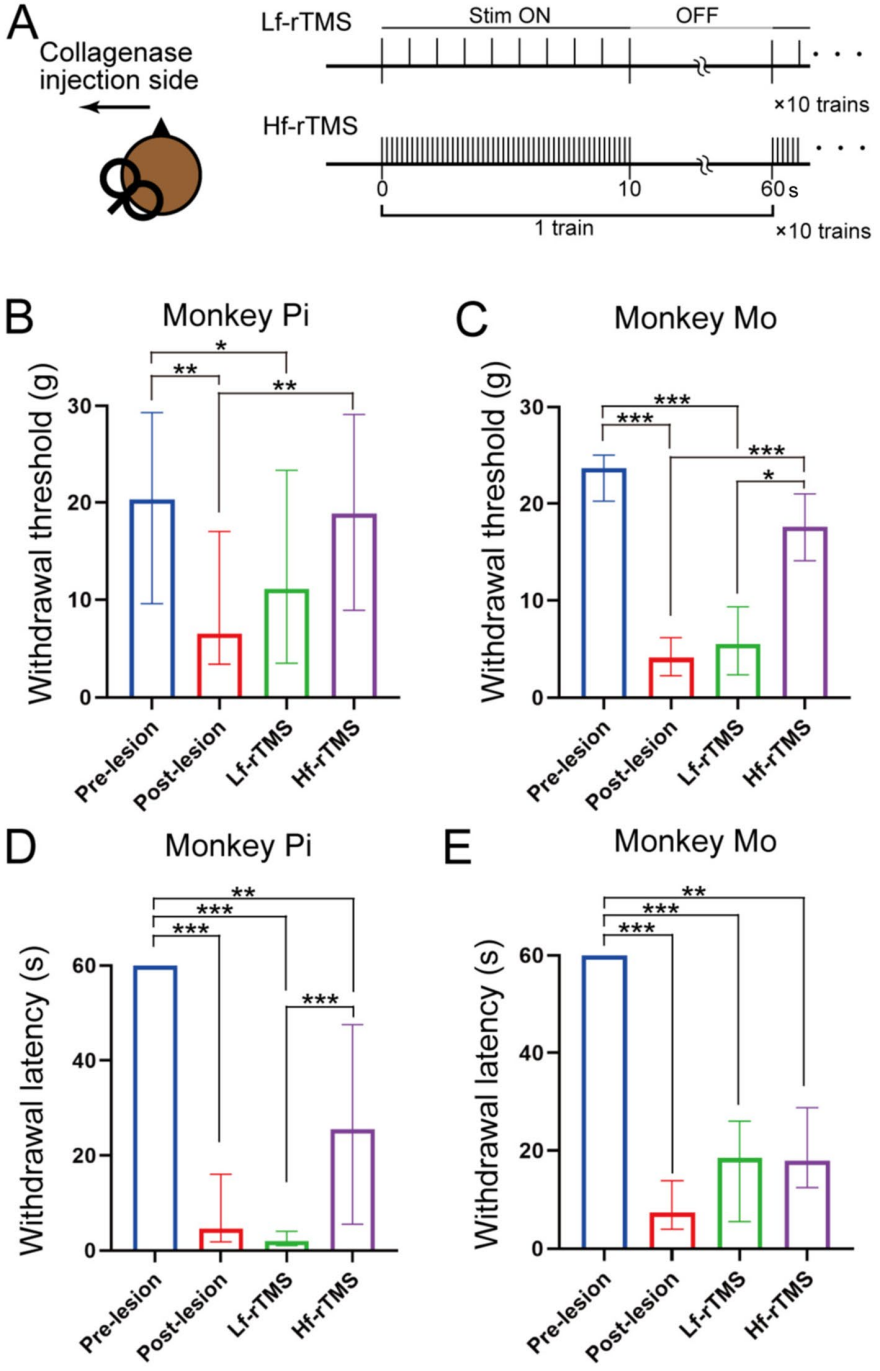
Effect of repetitive transcranial magnetic stimulation on withdrawal threshold. (**A**) rTMS protocol. Coils were placed in M1 on the side of collagenase injection and magnetic stimulation was administered at 1 or 5 Hz. (**B**, **C**) Changes in withdrawal threshold to mechanical stimulation. (**D**, **E**) Changes in withdrawal latency to thermal stimulation (50 °C). Data are expressed as median and interquartile ranges. *p < 0.05, **p < 0.01, ***p < 0.001 (Mann–Whitney U test with Bonferroni correction followed by Kruskal–Wallis test).

Temporal changes in both monkeys’ mechanical and thermal withdrawal thresholds employed in the current experiment before and after the VPL lesion were reported in our previous publications^21,22,27^. This study also demonstrated withdrawal threshold alterations following Hf- and Lf-rTMS (Fig. 2A). In the mechanical withdrawal test, significant differences were observed between Monkeys Pi and Mo (Kruskal–Wallis test, *p* < 0.001 and *p* = 0.001, respectively) (Fig. 2B, C). A post hoc Mann–Whitney U test (with Bonferroni correction) revealed a significant decrease in the post-lesion and Lf-rTMS condition thresholds compared with the pre-lesion values. Moreover, in both monkeys, a significant increase in the threshold was observed after Hf-rTMS compared to the post-lesion values. The thresholds were significantly higher in Monkey Mo than in the Lf-rTMS condition. In the thermal withdrawal test, significant differences were also observed between the conditions in both monkeys (Kruskal–Wallis test, *p* < 0.001) (Fig. 2D, E). The pre-lesion withdrawal latency was significantly longer than the other conditions in both monkeys. In Monkey Pi, the withdrawal latency was significantly prolonged with Hf-rTMS compared to Lf-rTMS. To summarize the results for both monkeys, the thresholds for mechanical stimulation after the Hf-rTMS were higher than those in the post-lesion condition. The withdrawal latency to thermal stimulation also showed an increasing trend after Hf-rTMS.

### fMRI

**Figure 3:**
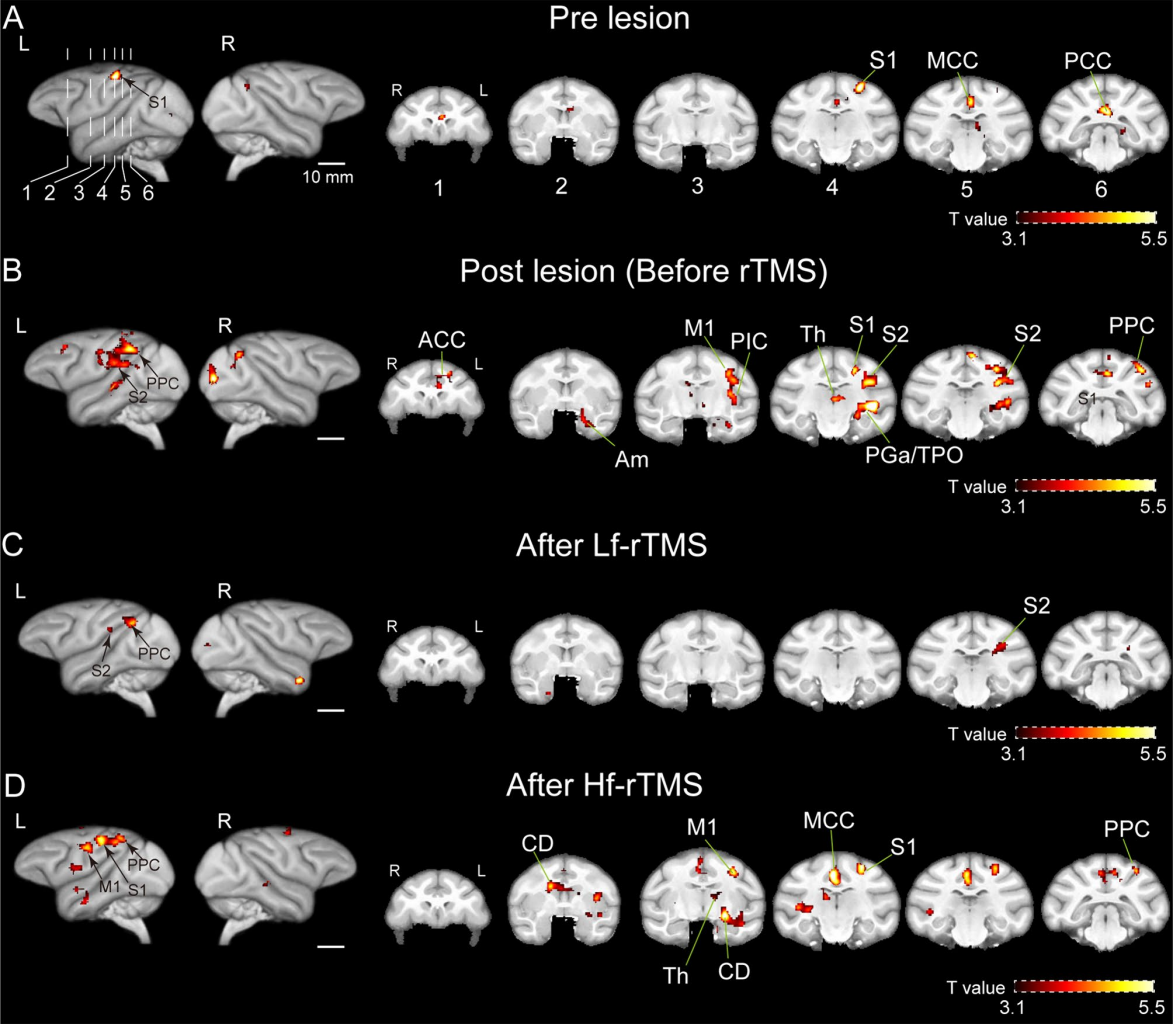
Somatosensory activity changes associated with rTMS. (**A**–**D**) Brain activity associated with cutaneous tactile stimulation to the right hand compared with that to the left hand in the pre-lesion condition (**A**), post-lesion condition (before rTMS) (**B**), after Lf-rTMS (**C**), and after Hf-rTMS (**D**). Statistical significance was set at *p* < 0.05 FWE cluster correction (primary clustering threshold *p* < 0.001, uncorrected). The statistical t-maps were superimposed on the macaque template^52^, and the t-values are presented on a color scale. ACC, anterior cingulate cortex; Am, amygdala; CD, caudate nucleus; MCC, midcingulate cortex; M1, primary motor cortex; PGa, anterior subdivisions of the angular gyrus; PIC, posterior insular cortex; PPC, posterior parietal cortex; S1, primary somatosensory cortex; S2, secondary somatosensory cortex; Th, thalamus; TPO, temporoparietooccipital junction; rTMS, repetitive transcranial magnetic stimulation; Lf, low frequency; Hf, high frequency; FWE, family-wise error.

fMRI analysis was performed to examine the changes in brain activity before and after thalamic lesions and the rTMS intervention. Figure 3A shows the brain activity response to the right-hand cutaneous tactile stimulation (corresponding to the contralesional hand) before the thalamic lesion. As in many previous studies using fMRI to analyze tactile stimulation of monkey hands, significant activation related to tactile stimulation was confirmed in the left hemisphere S1, midcingulate cortex (MCC), and posterior cingulate cortex (PCC) (family-wise error [FWE] cluster-corrected *p* < 0.05)^22,28–31^. The monkeys demonstrated stable decreased withdrawal thresholds reflecting allodynia and hyperalgesia five months after the thalamic lesions were induced; significant activation was observed in pain-related brain regions such as the ACC, thalamus, posterior parietal cortex (PPC), posterior insular cortex (PIC), S2, and S1 in the left hemisphere, which was the ipsilesional hemisphere (FWE cluster-corrected *p* < 0.05) (Fig. 3B). After Lf-rTMS, the regions that showed significant activation were the S2 and PPC of the left hemisphere (FWE cluster-corrected *p* < 0.05) (Fig. 3C). In contrast, after Hf-rTMS, significant activation was found in the S1, M1, MCC, and PCC in the left hemisphere (FWE cluster-corrected *p* < 0.05) (Fig. 3D). The statistics for the clusters and other activation areas are presented in Table 1.

**Table 1:** Regions of brain activation.

Cluster size (voxels)	Cluster *p*-value (FWE)	Local maxima (side of hemisphere)	t-value	Coordinates (x, y, z)
Pre-lesion				
113	0.009	S1 (Left)	6.09	12, − 13, 19
365	< 0.0001	MCC	6.44	0, 2, 12
400	< 0.0001	PCC (Left)	5.90	1, − 21, 8
133	0.004	V1 (Left)	5.62	15, − 34, 3
Post-lesion (before rTMS)				
2075	< 0.0001	PGa (Left)	5.89	19, − 13, − 3
		PPC (Left)	5.43	19, − 22, 15
		S2 (Left)	5.15	16, − 15, 8
89	0.022	S1 (Left)	5.33	4, − 17, 21
79	0.036	PPC (Right)	4.71	− 17, − 30, 13
124	0.005	PCC (Left)	4.69	3, − 20, 13
118	0.006	ACC (Left)	4.46	4, 10, 10
282	< 0.0001	V1 (Right)	6.29	− 10, − 42, 2
After Lf-rTMS				
100	0.013	S2 (Left)	3.93	15, − 15, 10
94	0.018	PPC (Left)	4.78	14, − 26, 13
83	0.030	TGa (Right)	5.45	− 11, 2, − 13
80	0.034	V1 (Right)	4.46	− 7, − 37, 2
After Hf-rTMS				
1321	< 0.0001	MCC (Right)	5.68	− 2, − 14, 14
		S1 (Left)	5.19	12, − 14, 18
		PCC (Right)	5.01	− 1, − 23, 17
521	< 0.0001	CD (Left)	5.29	11, − 8, − 6
		TPO (Left)	4.08	18, − 6, − 11
		Th (Left)	4.02	7, − 10, 4
348	< 0.0001	CD (Right)	5.18	− 6, 0, 10
		CD (Left)	3.41	7, 0, 5
164	0.001	ACC (Left)	4.83	2, 12, 9
103	0.012	M1 (Left)	4.82	14, − 7, 14
195	< 0.0001	Pu (Left)	4.58	17, − 5, 5
185	< 0.0001	TPO (Right)	4.39	− 18, − 14, − 1

Coordinates are in mm. ACC, anterior cingulate cortex; Am, amygdala; CD, caudate nucleus; FWE, family-wise error; Hf-rTMS, high-frequency repetitive transcranial magnetic stimulation; M1, primary motor cortex; Lf-rTMS, low-frequency repetitive transcranial magnetic stimulation; PCC, posterior cingulate cortex; PGa, anterior subdivisions of the angular gyrus; PPC, posterior parietal cortex; Pu, putamen; S1, primary somatosensory cortex; S2, secondary somatosensory cortex; TGa, agranular part of the temporal pole, Th, thalamus; TPO, temporo-parieto-occipital junction; V1, primary visual cortex.

**Figure 4:**
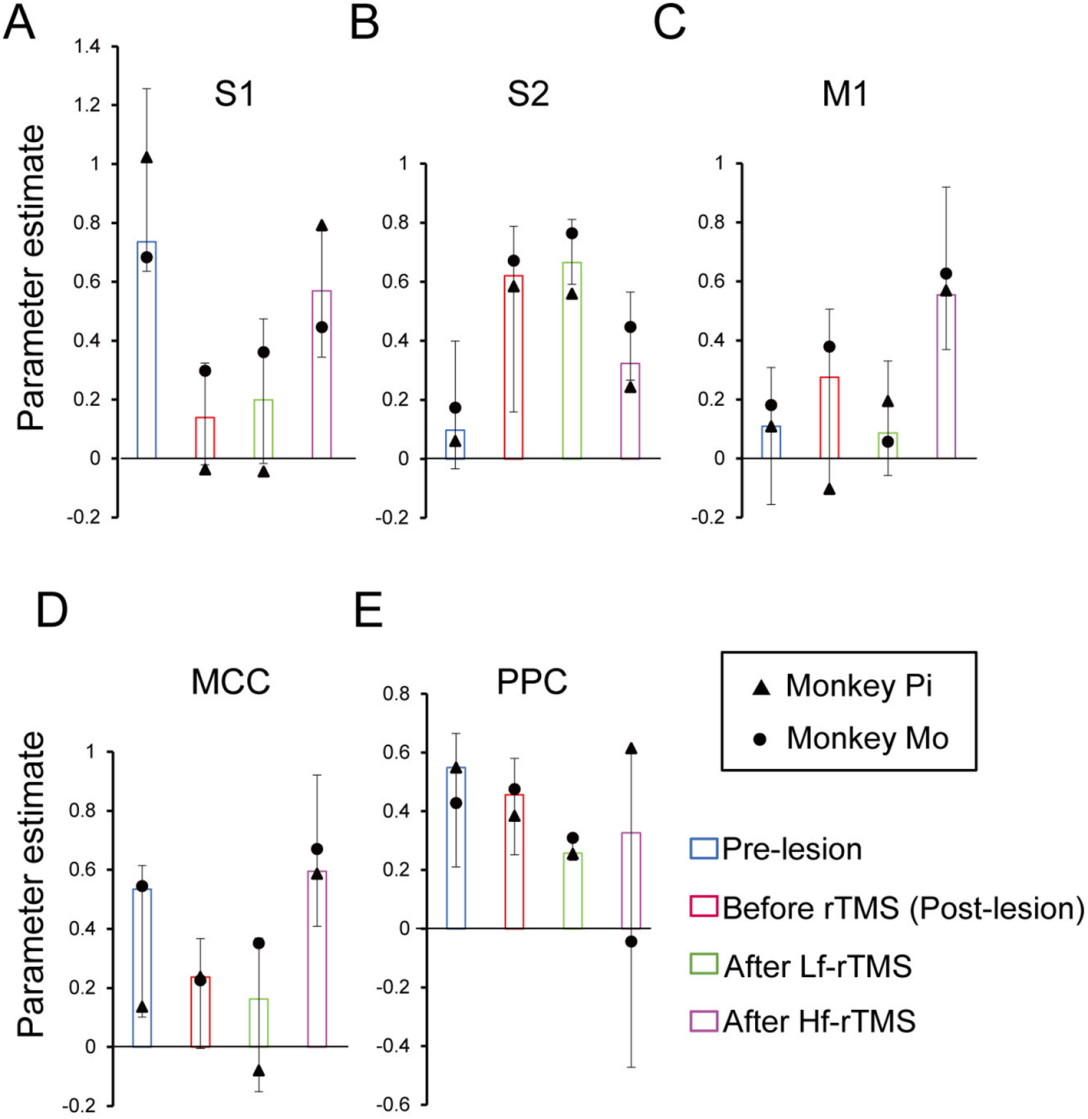
Degree of somatosensory activity in each condition. (**A**–**E**) Median of beta estimate values for the four conditions. The error bars in the graph indicate the interquartile range for all datasets in the condition.

We investigated the degree of sensory fMRI signals across conditions by strategically placing regions of interest (ROIs) and beta estimate values were derived for each individual. Functional ROIs in the S1, M1, MCC, and PCC were calculated using thresholded t-maps (uncorrected *p* < 0.001) obtained from the Hf-rTMS conditions, defined as continuous voxels with a 2-mm radius centered at the local maximum. In addition, the functional ROI in the S2 was calculated using thresholded t-maps (uncorrected *p* < 0.001) obtained from the Lf-rTMS conditions. The voxel coordinates of each ROI are listed in Table 2. Figure 4 shows the median beta estimates calculated for each functional ROI. In the S1, sensory-evoked activity decreased following thalamic lesions (Fig. 4A). This reduced activity remained unaltered after Lf-rTMS but tended to increase after Hf-rTMS in both monkeys. The S2 exhibited higher activity after the lesion than before, with this elevated activity remaining unchanged after Lf-rTMS but showing a tendency to decrease after Hf-rTMS (Fig. 4B). The M1 and MCC showed increased activity after Hf-rTMS compared with the other three conditions (Fig. 4C, D). The PCC showed a similar degree of activity before and after the lesion but slightly decreased after Lf-rTMS; each monkey had a different activity after Hf-rTMS.

**Table 2:** ROI information.

Region	Coordinates of the central voxel (x, y, z)	
	Monkey Pi	Monkey Mo
S1	12, − 13, 19	11, − 13, 18
S2	11, − 16, 7	16, − 15, 8
M1	14, − 8, 16	15, − 9, 14
MCC	− 1, − 14, 14	0, − 14, 11
PPC	16, − 20, 18	19, − 18, 14

Coordinates are in mm. ROI, region of interest; S1, primary somatosensory cortex; S2, secondary somatosensory cortex; M1, primary motor cortex; MCC, midcingulate cortex; PPC, posterior parietal cortex.

## Discussion

In this study, using a CPSP monkey model^21^, we demonstrated that Hf-rTMS elevated mechanical and thermal stimuli withdrawal thresholds, whereas Lf-rTMS failed to induce threshold alterations. Furthermore, sensory fMRI showed that activation of the ipsilesional S2 was a cortical focus reflecting abnormal pain perception.

Detailed behavioral test time-series data in CPSP model monkeys have been described previously^21,27^. Notably, the latency between collagenase injection into the VPL and the onset of behavioral changes reflecting CPSP development is several weeks, similar to humans with CPSP^32,33^. In addition, we used fMRI to examine changes in brain activity in response to tactile stimulation after a thalamus lesion and found activation of brain regions such as the thalamus, PIC, S2, and ACC, similar to that observed in patients with CPSP^3,10,34,35^. The differences in analgesic effects according to stimulation frequency observed in the present study are also similar to those in patients with pain symptoms^7,10,13,15,36^. This experiment was designed to determine only immediate changes in brain activity after rTMS. Thus, future studies should aim at identifying reveal long-term effects to establish an optimal rTMS intervention.

Through fMRI analysis, we demonstrated that when behavioral results reflecting CPSP are observed, S1 activity in response to tactile stimulation was reduced compared to that before the thalamic lesion. This decrease was also similar to that reported in the results of an electroencephalography study in patients with CPSP^5^. This may be due to subthalamic nuclei damage, including the VPL, which conveys somatosensory information from the periphery to sensory-related cortical areas^37^. The reduced S1 activity increased after Hf-rTMS, whereas no such changes occurred after Lf-rTMS. In patients, fiber tracking imaging indicated that good delineation of the thalamocortical pathway was associated with the analgesic effect of Hf-rTMS targeting the M1^10,38^. This evidence suggests the importance of preserving thalamocortical pathways after stroke and reactivating the S1 for the analgesic effect of Hf-rTMS.

**Figure 5:**
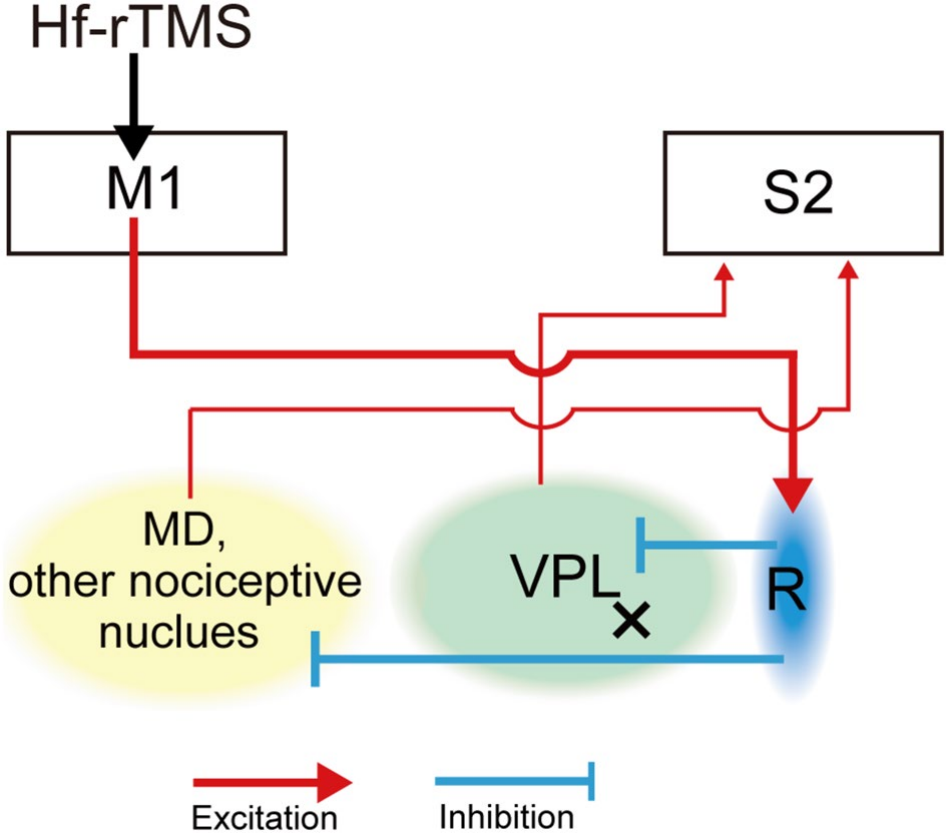
Hf-rTMS-induced decrease in S2 activity via the cortico-thalamo-cortical pathways. Hf-rTMS increases M1 excitability, which in turn increases neurotransmission to the R neurons. The R neurons have been shown to inhibit thalamic relay neurons, including nociceptive neurons in the MD, which could eventually lead to reduced activation of S2. MD, mediodorsal nucleus; R, reticular thalamus.

The present fMRI results support previous studies showing that the degree of S2 activation in somatosensory processing by CPSP is associated with that of analgesia induced by rTMS^10^. Indeed, our previous studies have shown that the local pharmacological inhibition of S2 in CPSP model monkeys transiently alleviates allodynia and hyperalgesia without completely inhibiting physiological tactile and pain perception^22,27^. The inhibition of S2 that produces analgesia may be mediated through cortico-cortical or cortico-thalamo-cortical pathways from M1. For example, focusing on the cortical-thalamo-cortical pathway, the increased excitability of M1 by Hf-rTMS may activate its projection site, the reticular thalamic nucleus^39^. The neurons in the reticular nucleus have been shown to inhibit thalamic relay neurons, including nociceptive neurons in the thalamic mediodorsal nucleus (MD)^40^, which would eventually lead to reduced activation of S2 (Fig. 5)^41,42^. Mesoscopic brain mapping using fMRI did not provide a detailed picture of the neural circuits contributing to Hf-rTMS-induced analgesia. To verify the neural circuits responsible for the analgesic effect of Hf-rTMS, it will be important for future studies to obtain evidence of neural activity using single-unit recordings from each region after M1-rTMS. Also, unit single-recordings will reveal whether cortico-cortical pathways involving inhibitory neurons within the cortex are involved in the analgesic effects. While it is necessary to conduct invasive experiments using the monkey model, the outcomes will contribute to a better understanding of interventions for patients with CPSP.

Including the above view, it has been hypothesized that rTMS modulates individual areas and regionwide networks^7,23^, contributing to analgesia. The changes in MCC and PCC activation observed in the Hf-rTMS condition in the present study likely reflect sensory processing changes due to their anatomical connections with regions such as the S1 and M1^29,43^. In addition to the cortical network, changes in various subcortical region activity, including the caudate nucleus, amygdala, and thalamus, may also reflect modulation in sensory networks induced by rTMS targeting the M1, in any case supporting the network hypothesis. Understanding how functional networks involving these regions contribute to analgesia will elucidate the mechanism of this effect. For instance, modulating local activity (e.g., through local muscimol injection) prior to rTMS may aid in identifying brain regions that are causally related to analgesia. A crucial question in such a validation is whether the experimental animals have a neural network comparable to that of humans. Monkeys have relatively large brains compared to rodents, and their somatosensory-related anatomical connection patterns are similar to those of humans^37^. These facts suggest that studies using monkey models may contribute to capturing the anatomical basis or functional changes related to underlying analgesia obtained by brain imaging after rTMS interventions in human patients^10^.

## Methods

### Subjects

Two rhesus macaques (*Macaca mulatta*; Macaque Mo: male, 5.1 kg; Macaque Pi: male, 8.9 kg) were used in this study. The behavioral data and specific MRI data in the present study were originally acquired during previous studies^21,22,27,44^. The Animal Care and Use Committee of the National Institute of Advanced Industrial Science and Technology approved this experiment, and the animal use protocol was carried out in accordance with the guidelines within the “Guide for the care and Use of Laboratory Animals” (Eighth ed., National Research Council of the National Academies). They are also in accordance with ARRIVE guidelines. As previously described^21^, two MR-compatible head posts were implanted in the monkeys under anesthesia and in sterile surgical conditions. The head posts were embedded in the dental acrylic that covered the top of the skull, and they were connected to the skull using non-magnetic screws.

### Collagenase injection and estimation of lesion area

As previously described^21^, we created a CPSP model based on hemorrhagic lesions in the thalamus’s left VPL. Briefly, the area of the left VPL activated by somatosensory stimulation of the right hand was identified electrophysiologically and collagenase type IV (C5138; Sigma, St. Louis, MO; 200 U/mL in saline) was injected into the region. Both monkeys received two separate 4-µL injections spaced 1 mm apart in the dorsoventral direction.

The extent of the hemorrhagic lesions were identified by histological experiments, as described previously^21^.

### rTMS protocol

The rTMS intervention was performed three months after the VPL lesion. During the post-lesion period, monkeys exhibit stable behavioral changes that reflect CPSP^21,22,27^. Single-pulse stimulations were administered to identify the hand region of the left M1 and active motor threshold, defined as the value at which the stimulus moved the monkey’s right hands with an approximately 50% probability^23^. We used a MagStim Rapid^2^ stimulation system (Magstim, Whitland, UK) connected to a figure-eight coil or double cone coil (Magstim)^45^. The coil was held laterally at an angle of approximately 50° to the sagittal plane, with the handle pointing backwards. rTMS was applied at 90% motor threshold intensity, and the monkeys received trains of 10 s at 5 Hz (defined as Hf-rTMS) or 1 Hz (defined as Lf-rTMS). The trains were repeated 10 times, and the interval of each train was 50 s. Therefore, 500 and 100 pulses were applied under Hf- and Lf-rTMS conditions, respectively (Fig. 2A). During all rTMS interventions, the coils were covered with clay and the skull with dental acrylic to prevent misalignment.

### Behavioral tests

Mechanical and thermal withdrawal tests were performed^21^. The mechanical withdrawal test was conducted using an electronic von Frey anesthesiometer (IITC Life Science, Woodland Hills, CA). Each von Frey filament was applied perpendicularly to the palmar surface of the second, third, and fourth fingers, and the maximum pressure that triggered hand withdrawal was assessed. Two trials were performed for each finger, and the higher pressure was adopted as the datum for that day.

Thermal withdrawal tests were performed using a thermal plate (SCP-85; As One Corporation, Osaka, Japan) maintained at 50 °C. The monkey’s hand was placed on the plate under conditions that allowed free withdrawal from the thermal stimulus. The degree to which the thermal stimulus affects withdrawal latencies was evaluated by measuring thermal stimulation latencies to avoid exceeding the upper limit of 60 s, which accounts for low-temperature burn injury. Up to five trials were performed at 50 °C until the plate contact time exceeded 90 s. Hand withdrawal latencies were estimated in real-time during the test.

### Behavioral analysis

The behavioral test captured the movements of both hands using two independent digital video cameras (HC-V520M; Panasonic, Osaka, Japan) placed around the task apparatus. A subset of the recorded videos was later reanalyzed by an individual unaware of the intervention. The three trials immediately before VPL lesion induction were treated as pre-lesion behavioral data. The rTMS intervention period was 20–23 and 12–19 weeks post-lesion for Monkeys Pi and Mo, respectively. Data from the three days immediately preceding the start of the intervention period were used as post-lesion data for each monkey. rTMS interventions were administered once daily for three days each in a randomized order. Therefore, each condition comprised three days of behavioral data. Each intervention was spaced three days apart to eliminate cumulative rTMS effects over time^23^. The Kruskal–Wallis test followed by the Mann–Whitney U test with Bonferroni correction were performed to assess the difference in withdrawal threshold and latency between each condition. Statistical significance was set at *p* < 0.05.

### MRI scanning

Anatomical MRI and fMRI scans were conducted before and five months after collagenase injection (Monkey Pi: 24–28 weeks; Monkey Mo: 20–23 weeks after injection) using a 3.0-T MRI system (Philips Ingenia 3.0 T, Philips Healthcare, Best, the Netherlands). The anatomical MRI included a T1-weighted image (repetition time [TR]/echo time [EPI], 7.3/3.2 ms; number of excitations, 2; flip angle, 8°; field of view, 134 × 134 mm; matrix, 224 × 224; slice thickness, 0.6 mm; number of slices, 200). fMRI was acquired via field–echo echo–planar imaging (EPI; TR/TE, 3,000/35 ms; flip angle, 90°; field of view, 120 × 120 mm; matrix, 64 × 64; in-plane voxel dimensions, 1.875 × 1.875 mm; slice thickness, 2.2 mm; spacing between slices 2.2 mm; number of slices, 27)^22^.

Before scanning, the monkeys were sedated through continuous intravenous infusion of propofol (0.4 mg/kg/min), with a slight analgesic effect, if any at all^46,47^. The monkey’s head was immobilized in the scanner. For post-lesion data acquisition, fMRI was initially performed without rTMS (“Before rTMS”). After fMRI, the monkeys were returned to wakefulness by ceasing the continuous propofol administration. Approximately 1 h after ceasing the propofol, when the monkeys were awake, Hf- or Lf-rTMS was administered as described in the rTMS protocol section. Anesthesia was resumed after completing the last pulse stimulation, and the animals were returned to the MRI scanner. fMRI scanning was performed after rTMS, and this measurement was completed 2 h after the completion of rTMS.

A block-design paradigm was used in the fMRI to measure the brain response to somatosensory tactile stimulation^22,29,30,48^. Specifically, a gentle tactile stimulus was applied to either the right or left hand for 30 s using an MR-compatible brush. During the stimulation block, stimuli were delivered at approximately 1 Hz to the monkey’s hands^22^. The stimulation block was followed by a 30-s no-stimulus period and subsequent stimulation of the opposite hand similarly. A single scanning run comprised five or ten stimulation blocks for both hands, with two to four runs daily. In total, 80 and 160 stimulus blocks were acquired in the pre-lesion period (Monkey Pi, 40 blocks in eight runs; Monkey Mo, 40 blocks in four runs) and before rTMS (80 blocks in eight runs for each monkey), respectively. In addition, 80 stimulus blocks were acquired after Hf- and Lf-rTMS (40 blocks in 4 runs for each monkey). Each MRI was spaced three days apart to eliminate cumulative effects^23^.

### fMRI analysis

Preprocessing for fMRI analysis was almost identical to that used in our previous studies^22,49^. The preprocessing steps were performed using SPM12 software (Wellcome Department of Cognitive Neurology, London, UK). Briefly, the acquired EPIs underwent motion correction and spatial normalization to a macaque brain template^34^, and were resliced to a voxel size of 1 × 1 × 1 mm. The resulting images were smoothed using a Gaussian kernel of 3 × 3 × 3 mm (full width at half maximum).

Sensory fMRI signals were investigated using a fixed-effects general linear model to analyze the block data. This modeling strategy is frequently employed in brain imaging studies in monkeys, particularly in situations where a restricted number of macaques are used because of practical limitations^30,50,51^. Consistent with our previous study^22^, the contrasts involved comparing the right-hand stimulation (the hand contralateral to the collagenase-injected hemisphere) without left-hand stimulation. The initial voxel threshold was set at an uncorrected *p* < 0.001. The cluster’s significance was determined at *p* < 0.05 corrected for FWE. The resulting t-maps were overlaid onto the macaque brain template^52^ using MRIcro software (http://www.mricro.com).

To examine sensory-evoked activation in the regions identified as highly significant in the above sensory fMRI results, we calculated the beta estimate values of each ROI in each monkey. Spherical ROIs of 2-mm radius were created using the MarsBar toolbox (http://marsbar.sourceforge.net), and the coordinates of the ROI center were taken from the significant peak voxels observed in the fMRI results for each monkey (uncorrected *p* < 0.001).

## Data Availability

The datasets generated during the current study are available from the corresponding author (Kazuaki Nagasaka at nagasaka@nuhw.ac.jp) on reasonable request.
